# Biological Characteristics of Experimental Genotype Mixtures of Cydia Pomonella Granulovirus (CpGV): Ability to Control Susceptible and Resistant Pest Populations

**DOI:** 10.3390/v8050147

**Published:** 2016-05-21

**Authors:** Benoit Graillot, Sandrine Bayle, Christine Blachere-Lopez, Samantha Besse, Myriam Siegwart, Miguel Lopez-Ferber

**Affiliations:** 1 LGEI, Ecole des Mines d’Alès, Institut Mines-Telecom et Université de Montpellier Sud de France, 6 Avenue de Clavières, 30319 Alès, France; graillot.benoit@gmail.com (B.G.); sandrine.bayle@mines-ales.fr (S.B.); christine.blachere-lopez@mines-ales.fr (C.B.-L.); 2 Natural Plant Protection, Arysta LifeScience Group, Avenue Léon Blum, 64000 Pau, France; samantha.besse@arysta.com; 3 INRA, 6, Avenue de Clavières, 30319 Alès, France; 4 INRA, unité PSH, Agroparc, 84914 AVIGNON Cedex 9, France; myriam.siegwart@avignon.inra.fr

**Keywords:** CpGV, Lepidoptera, genetic heterogeneity, resistance, biological control

## Abstract

The detection of resistance in codling moth (*Cydia pomonella*) populations against the Mexican isolate of its granulovirus (CpGV-M), raised questions on the sustainability of the use of this biological insecticide. In resistant host cells, CpGV-M is not able to complete its replication cycle because replication is blocked at an early step. Virus isolates able to overcome this resistance have been characterized—among them, the CpGV-R5 isolate. In mixed infections on resistant insects, both CpGV-M and CpGV-R5 viruses replicate, while CpGV-M alone does not induce mortality. Genetically heterogeneous virus populations, containing 50% of each CpGV-M and CpGV-R5 appear to control resistant host populations as well as CpGV-R5 alone at the same final concentration, even if the concentration of CpGV-R5 is only half in the former. The use of mixed genotype virus preparations instead of genotypically homogeneous populations may constitute a better approach than traditional methods for the development of baculovirus-based biological insecticides.

## 1. Introduction

The Cydia pomonella granulovirus Mexican isolate (CpGV-M) (species *Cydia pomonella granulovirus*; genus *Betabaculovirus*; family *Baculoviridae*) [1] was discovered by Tanada in 1964 [2]. This virus has a narrow host range, limited to the codling moth (*Cydia pomonella*) and some related moth species. The CpGV-M genome comprises double stranded circular DNA of 123.5 kb in length [3]. CpGV has been registered as a biocontrol agent in different countries to control the codling moth in apple and pear orchards. In Europe, most commercial formulations of CpGV are derived from the original CpGV-M isolate [4], which has been distributed to many different laboratories and companies, and amplified on laboratory colonies of codling moth. This isolate appears to have limited genetic diversity [5].

Resistance to CpGV-M was first reported in Germany in 2005 [6], and subsequently in France [7]. Recent reports indicate a wide distribution of CpGV-resistant populations across Europe [8] but not in other continents. The resistance observed in all Europe appears to have a similar genetic origin. The resistance determinant (gene) is located in the sex chromosome of the host and is dominant. Resistant females are Z^R^W, while resistant males can be Z^R^Z^R^ or Z^R^Z^S^ [9]. The mechanism of resistance, analyzed on the laboratory colony CpRR1, appears to involve early blocking of viral replication in host cells [10].

Research was conducted to obtain new viral variants able to overcome the resistance mechanisms of host cells. Extensive collections of CpGV isolates were carried out in the original apple distribution area [11]. New isolates able to control resistant populations were obtained by selecting isolates from laboratory and field collections that were capable of lethal infection of resistant codling moth larvae [12,13], proving that an important degree of genetic variability is present in CpGV populations and that this variability is reflected in biological (phenotypic) differences. The inability of CpGV-M to successfully replicate in resistant hosts is linked to a modification of the *pe38* gene, the function of which remains unclear [14].

Both resistant and susceptible individuals are usually present simultaneously in field host populations and this raises questions on the ability of a single viral genotype to provide adequate control of the pest population, and the risk of the development of resistance to other genotypes in the virus population.

Genetic diversity in parasite populations is an important driver for the evolution of host defense capabilities [15,16]. Natural virus populations are genetically heterogeneous and certain genotypic variants may be more prevalent than others [17,18]. The fact that minority genotypes are not eliminated in wild populations suggests that this heterogeneity is important for virus survival [19].

As pathogens and their hosts are continuously coevolving, it was thought that pest populations were unlikely to develop resistance to a pathogen-based product [20], contrary to the resistance observed with chemical insecticides [21]. The development of insect pathogens for use in inundative biological control has relied on this assumption for planning the timing and frequency of application of virus Occlusion Bodies (OBs). However, Briese *et al.* [22] noted that if selection for resistance was possible in the laboratory, the same could occur in natural pest populations subjected to continuous exposure to the virus.

A major question arising is why resistance to CpGV spread recently in apple and pear agrosystems and has not been detected in natural populations of codling moth. Fuxa pointed out the importance of the ability of the virus to adapt and therefore overcome resistance of the host in natural conditions [23]. Possible answers might involve differences between the genetic homogeneity of virus-based insecticides over space and time and the diversity of natural isolates. The genotypic diversity present in the virus population at a given moment comprises variation in geographical diversity and temporal diversity with changes in the relative frequency or identity of virus genotypes over time.

Genetic variability has been described both in nucleopolyhedroviruses (NPVs, family *Baculoviridae*, genus *Alphabaculovirus*) [24,25,26] and in granuloviruses (GV) populations [5,27,28]. In recent years, a renewal of interest in the role of genetic diversity has led to studies on the genetic structure of NPV wild populations [25] and the mechanisms involved in generating and maintaining such diversity. The phenomenon of co-infection of host cells has been studied and shown to be a generalized trait in Autographa californica multicapsid nucleopolyhedrovirus (AcMNPV) [29]. Co-occlusion, *i.e.*, the possibility of transmission of more than one genotype in a single OB, has been demonstrated for Spodoptera frugiperda multiple nucleopolyhedrovirus (SfMNPV) [30,31]. Genetic variability in NPVs seems to be higher than in GVs. This was partially attributed to differences in the physical structure of OBs. In NPVs, a single OB contains multiple virions, which can represent the whole population diversity. In contrast, in GVs, a single OB contains a single virion. Consequently, infection following ingestion of one OB might result in a genotypically diverse infection in the former, and to a clonal infection in the later.

In the virus-host system SfMNPV and *Spodoptera frugiperda*, Simon *et al.* have shown that genetic heterogeneity contributes to the efficacy of the viral population by increasing the transmissibility of virus OBs [32], and that genetically diverse experimental populations are more effective insecticides than any of the individual component genotypes alone. In this manuscript, we have tested this approach with CpGV in susceptible and resistant populations of the codling moth.

## 2. Materials and Methods

### 2.1. Insects

Two laboratory colonies were used in this study: the susceptible colony CpNPP, used for the industrial production of CpGV by Natural Plant Protection SA (Pau, France), is susceptible to both CpGV-M and CpGV-R5 virus isolates. CpNPP has been reared in the laboratory for more than 25 years and originally comes from northern France.

The resistant colony R_GV_ of *Cydia pomonella* was derived from a natural resistant population (St-A) found in the region of Saint-Andiol (Bouches-du-Rhône, France) as previously described [13]. The R_GV_ colony harbors the major genetic determinant for resistance that is present in resistant European populations [13]. As this gene is located on the Z sex chromosome of the insect, and the induced phenotype is dominant [9], and using our approach of mass selection, it is impossible to completely eliminate the susceptible allele, as heterozygous males (Z^R^Z^S^) are resistant to CpGV-M infection. Accordingly, the R_GV_ colony is periodically subjected to artificial selection by rearing the survivors of a CpGV-M treatment in order to maintain a high level of resistance [13].

Both colonies are mass reared on artificial diets as previously described [13].

### 2.2. Viruses

The virus isolates used in this work, CpGV-M (laboratory stock 2020-s1) and CpGV-R5 (laboratory stock 2016-r16) have been described previously [33]. CpGV-R5 is able to overcome the resistance of the R_GV_ colony and produces a productive lethal infection in resistant insects. No genetic polymorphism has been detected in this virus isolate [33].

### 2.3. Mixed Viral Populations

Five mixed virus populations were constructed by mixing OBs resulting from infections of pure isolates CpGV-M and CpGV-R5 propagated on permissive CpNPP larvae. The proportions of each isolate in the mixed virus populations were 99% CpGV-M + 1% CpGV-R5; 95% CpGV-M + 5% CpGV-R5; 90% CpGV-M + 10% CpGV-R5; 50% CpGV-M + 50% CpGV-R5; 10% CpGV-M + 90% CpGV-R5. Pure CpGV-M and CpGV-R5 were used as control populations. These OBs are referred as passage zero OBs (P0).

### 2.4. Amplification of the Different Viral Mixed Populations

Amplification of the different P0 populations was performed as previously described [33,34]. Briefly, third-instar (L3) susceptible or resistant larvae were inoculated using 50 µL of a mixed genotype suspension at a concentration of 800 OBs/µL, which was deposited on the surface of a formaldehyde-free diet (Stonefly *Heliothis* Diet, Ward’s Science, Rochester, NY, USA) in 24-well plates. One L3 larva was then placed in each well, the plates sealed and incubated at 25 °C (±1 °C) with a 16:8 h (light/dark) photoperiod and a relative humidity of 60% (±10%). After 4 days, larvae showing clear signs of infection were removed from the wells and kept without diet at 25 °C for one more day. Infected larvae were then crushed in distilled water and the resulting mixture was filtered through nylon to eliminate debris. The suspension was then centrifuged 5 min at 8000× *g*, and the resulting pellet was resuspended in distilled water. The final volume of the virus stocks was of 100 µL per infected larva, which corresponds to 10^11^ OB/mL on average. This suspension constituted the first amplification (P1) of each viral mixture.

### 2.5. Bioassays

Bioassays were performed on virus populations, (P0), using established protocols [13]. Briefly, 96-well plates containing 200 µL of formaldehyde-free diet (Stonefly *Heliothis* Diet, Ward’s Science, Rochester, NY, USA) were inoculated by spreading 6 µL of an OB suspension over the surface of each piece of diet by pipetting. Diet in control wells was treated with the same volume of distilled water. One larva, aged less than 12 h, was placed in each well. Six CpGV concentrations from 2 to 6250 OBs/µL on fivefold serial dilutions were used in all but CpGV-M bioassays on resistant populations, in which concentrations between 10 and 3,000,000 OBs/µL were employed. Early mortality due to handling was excluded from the test. Virus induced mortality was recorded at 7 days post-infection. Mortality data were subjected to probit analysis [35] using the POLO + software [36]. To test the hypothesis of Independent Joint Action, the χ^2^ statistic was used following Polo Mix Software (LeOra Software) [36]. Each bioassay was repeated at least three times. Tests presenting a high mortality in controls (>10%) were rejected. The results for each treatment were pooled after verification of homogeneity.

### 2.6. Evaluation of the Relative Proportions of Each Genotype by PCR

#### 2.6.1. DNA Extraction

OB stocks were obtained as detailed in 2.4. Lysis of OBs was performed in order to extract viral DNA from the OB suspensions obtained previously (P0–P1). A volume of 100 µL of extraction buffer (0.2 M Na_2_CO_3_, 0.34 M NaCl, 0.02 M EDTA; pH = 10.5) was added to 100 µL of OB suspension (~1 × 10^7^ OBs/µL) and incubated 30 min at 37 °C. The suspension was neutralized with 20 µL 1 M HCl and 11 µL 10% (wt/vol) sodium dodecyl sulfate (SDS) was added. A 1 min centrifugation step at 13,000 rpm was performed and the resulting supernatant was diluted (1/100) in sterile distilled water before (polymerase chain reaction) PCR amplification.

#### 2.6.2. PCR

##### Identification of the Virus Genotype

Restriction endonuclease analyses indicated that both CpGV-M and CpGV-R5 were likely to be genotypically homogeneous [33]. CpGV-R5 has been completely sequenced (unpublished work). By comparing the published genome sequence of CpGV-M and CpGV-R5, regions presenting differences were mapped. Gebhardt and coworkers [14] demonstrated that the ability of CpGV isolates to replicate in CpRR1 resistant hosts is due to a modification of the viral *pe38* gene. Similarly to their results, the difference between CpGV-M and CpGV-R5 *pe38* genes resides in a 24 bp. Gebhart *et al.* [14] suggested that the difference corresponds to an insertion in CpGV-M. This difference occurs in a repeated region of the gene, which impedes to differentiate between the two genotypes by qPCR. Accordingly, a pair of classical PCR primers was designed CpGV 19003R (5′ ccggctgcagCGAGTCGAGCACCACCATTA 3′) and CpGV18705F (5′ cgcgggatccACGGTGTGTCATTAGCCACC 3′), the numbers refer to the nucleotide positions in NC_002816 CpGV-M sequence. CpGV ORF 24 runs counterclockwise. These primers amplify fragments of differing size in the two genotypes (295 pb for CpGV-M and 315 bp for CpGV-R5), making it possible to discriminate between them. A restriction site was added at the extremity of each primer; *Pst*I and *Bam*HI for CpGV-18705F and CpGV-19003R, respectively, to facilitate eventual cloning.

PCR was performed in a final volume of 50 µL, containing 2 µL of each primer (10 pmol/µL MWG-Eurofins), 2 µL of viral DNA (*ca.* 10 pg), 20 µL of 2.5X PCR Hotmastermix (5 PRIME GmbH, Hilden, Germany) and 24 µL water. The amplification conditions were as follows: a 3 min predenaturation at 94 °C, followed by 35 cycles of 15 s, 94 °C; 40 s, 65 °C and 1 min 30 s, 72 °C.

**Figure 1:**
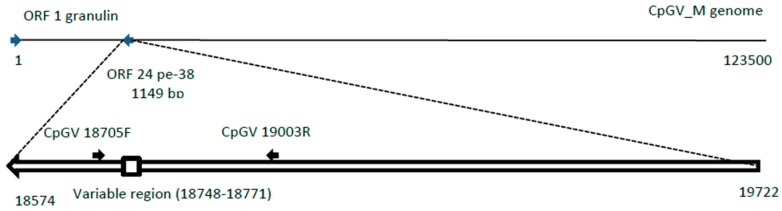
Schematic location of the PCR primers for amplification of the variable region as described in Reference [14]. Numbers refer to Cydia pomonella granulovirus Mexican isolate (CpGV-M) genome NC_002816 [3].

The amplified fragments were separated in a 3% agarose gel (NOVAGEL GQT, Conda S.A., Torrejon de Ardoz, Madrid, Spain) in tris-borate-ethylenediaminetetraacetic acid (TBE) buffer. Fragments were visualized on a UV transiluminator after ethidium bromide staining. (Figure 1).

## 3. Results

### 3.1. Efficacy of Viral Isolates

The concentration-mortality response of each viral population was estimated by probit analysis in our previous works [33,34] (Table 1).

CpGV-M was highly pathogenic towards the susceptible insect colony, CpNPP (Table 1, line 1), while even with high virus concentrations, the mortality on the R_GV_ resistant colony was very low. The LC_50_ value of CpGV-M OBs in R_GV_ larvae was 2.22 × 10^6^ OBs/µL, (Table 1, line 5). It has not been possible to calculate a LC_90_ value as R_GV_ population is not susceptible to this isolate. No mortality was observed when resistant R_GV_ larvae were inoculated with CpGV-M OBs at the standard concentration used for the amplification of the virus populations (800 OB/µL), as observed previously.

In contrast, CpGV-R5 was highly pathogenic to both insect colonies. The LC_50_ of CpGV-R5 alone ranged between 6.76 OBs/µL for the CpNPP colony (Table 1, line 4) and 22.43 OBs/µL for the R_GV_ colony (Table 1, line 8). For susceptible colonies, CpGV-R5 was as pathogenic as CpGV-M (compare Table 1, lines 1 and 4) [33,34].

For CpNPP susceptible insects, the LC_50_ observed for all virus populations tested varied between 6.76 and 13.10 OB/µL (Table 1, lines 1 to 4). The dose/response relationships although quite close, are statistically different (χ^2^ = 23.13, d.f. = 6, *p* < 0.01).

For the R_GV_ insect colony, the presence of CpGV-R5 genotypes, even at a low proportion (10%), results in a marked increase in pathogenicity compared to CpGV-M alone (measured both at the LC_50_ and LC_90_ levels) (Table 1, line 6).

For the mixed virus population, 50% CpGV-M + 50% CpGV-R5, the Independent Joint Action hypothesis was rejected both on susceptible and resistant insects, (Table 2), suggesting a positive interaction. However, when increasing the relative proportion of CpGV-M to 90% (M90%–R10% mix), the test did not reach the statistical signification threshold (α = 0.05) (Table 2).

### 3.2. Specificity of the Genotype Markers

**Figure 2:**
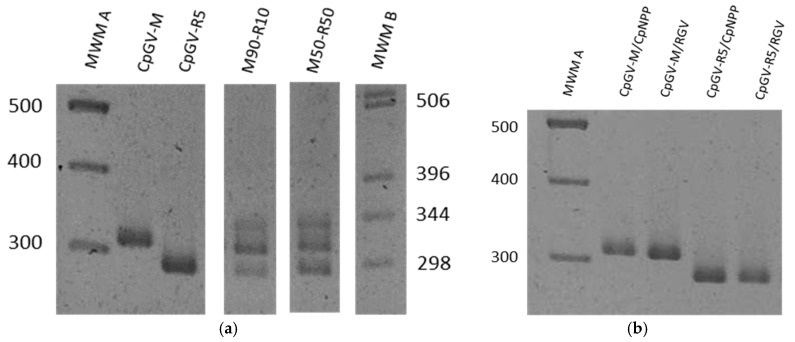
Gel electrophoresis of PCR products revealing the variability on the *pe38* region of Cydia pomonella granulovirus populations obtained by mixing isolates CpGV-M and CpGV-R5 on the proportions indicated (**a**) PCR of Occlusion Bodies (OBs) of pure virus isolates, and of mixed OBs; (**b**) PCR of OBs obtained after inoculation in both host colonies at 800 OB/µL in all but CpGV-M on R_GV_ that was inoculated at 30,000 OB/µL. MWM A: Molecular weight marker GeneRuler 100 bp DNA Ladder (Fermentas, Burlington, ON, Canada). MWM B: 1 kb DNA Ladder (Invitrogen, Carlsbad, CA, USA).

The suitability of the primers was tested both on the pure virus isolates CpGV-M and CpGV-R5 and on the laboratory mixed virus populations (P0). Single amplification fragments of the expected length were obtained for CpGV-M and CpGV-R5 pure isolates, confirming the specificity of the primers. For mixed P0 virus populations, both fragments were detected. (Figure 2a). Fragments of higher size were always found in the mixed OB extractions. As they appear before these OB replicate in the host, they cannot reveal a rearrangement, and must represent an artifact. They have not been analyzed further. No amplification was observed on PCR using uninfected larvae from the two colonies (data not shown).

### 3.3. Replication of Viral Genotypes in Different Hosts

OBs produced in both susceptible and resistant host colonies after infection with the experimental virus mixed populations were analyzed using this approach. Following infection of CpNPP and R_GV_ hosts with CpGV-R5, no CpGV-M markers were detected in the progeny OBs (Figure 2b). Similarly, in OBs produced in CpNPP following infection with CpGV-M, no CpGV-R5 markers were detected. Inoculation of R_GV_ larvae with CpGV-M at a concentration of 800 OB/µL did not result in virus-induced mortality. To verify the absence of persistent infection of R_GV_ or contamination of our CpGV-M virus stock with CpGV-R5, R_GV_ larvae were inoculated at a concentration of 30,000 OBs/µL. Even at this elevated concentration, no CpGV-R5 markers were detected in progeny OBs from the few (2 out of 24) larvae that subsequently developed obvious signs of infection.

**Figure 3:**
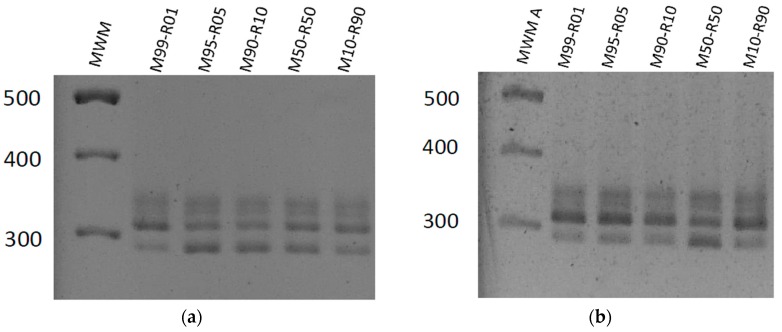
Gel electrophoresis of PCR products on P1 OBs obtained upon amplification of each virus mixed population. (**a**) on susceptible (CpNPP) larvae; (**b**) on resistant (R_GV_) larvae. MWM A: Molecular weight marker GeneRuler 100 bp DNA Ladder (Fermentas, Burlington, ON, Canada).

Markers corresponding to both CpGV-M and CpGV-R5 were detected in the progeny OBs of each mixture produced in CpNPP insects, indicating that both viruses were able to infect host cells, replicate and produce OBs (Figure 3a). Surprisingly, markers corresponding to both virus isolates were also detected in the viral progeny of each mixed virus population amplified in R_GV_ indicating that CpGV-M was able, in the presence of CpGV-R5, to infect resistant host cells and to replicate. (Figure 3b). Higher size fragments are observed. They are considered as artifacts, as on PCR of mixed P(0) OBs.

## 4. Discussion

On the susceptible colony CpNPP, both CpGV-M and CpGV-R5 alone can infect and kill the insects.

On the resistant colony R_GV_, the level of mortality obtained using pure CpGV-R5 was comparable to that of CpGV M on susceptible CpNPP colony (Table 1).

Using the R_GV_ colony, a lethal infection has been obtained by inoculating resistant insects with very high concentrations of CpGV-M (Table 1). However, no progeny viruses were recovered following inoculation of CpRR1 resistant insects with CpGV-M *per os* and by injection [10]. The difference in our results compared to the previously published results can be attributed to the dose range used. Asser-Kaiser and coworkers [10] carried out their test with a maximum concentration of 2 × 10^5^ OB/ mL of diet, while we have checked up to 2 × 10^8^ OB/mL of diet. Only when reaching concentrations higher than 10^6^ OB/mL did we start to observe the characteristic symptoms of infection by baculovirus in R_GV_ larvae. At concentrations of 800 OB/µL (that is, 2.4 × 10^4^ OB/mL of diet), CpGV does not induce mortality in resistant R_GV_ larvae. Although only R_GV_ larvae showing clear symptoms of infection were used for PCR, the amplification obtained using high virus concentration could be due to the inoculum OB that contaminated the larvae. Even in these conditions, no CpGV-R5 specific band was observed, as it would be expected if a latent infection was ongoing.

As CpGV-M does not induce mortality in resistant insects at the concentration of 800 OB/µL, using artificial mixed genotype preparations, we would expect a positive correlation between the relative proportion of CpGV-M in the inoculum and the dose required to kill larvae. This was clearly not the case. The presence of CpGV-M in the inoculum contributed to the overall pathogenicity of the mixed virus population. The markers we selected are linked to the ability to replicate in resistant hosts, recently demonstrated to reside in the *pe38* viral gene [14]. This means that a virus containing the “M” version of *p38* should not be able to productively infect a resistant insect. Given this scenario, we expected that no “M” type *pe38* would be present in the progeny OBs that replicated in R_GV_ insects. The presence of markers originating from both genotypes in the progeny of all mixed virus populations indicates that both viruses infected the larvae and replicated productively. The mechanism involved in the blocking of CpGV-M replication in resistant insects can be overcome, at least partially, by the presence of CpGV-R5.

Helper mechanisms can be classified in two main classes, those acting in *trans* and those acting in *cis*. Tanada and coworkers [37] first described baculovirus synergistic factors by analyzing the helper action of a granulovirus on a nucleopolyhedrovirus. They demonstrated that this effect occurred in *trans*; that is, one particle (an OB of a granulovirus) could help another particle (a virion of a nucleopolyhedrovirus) to initiate the infection of insect midgut cells. This action is due to the presence of a perithrophic membrane (PM) degradating enzyme present in the granulovirus OBs [38]. A different blocking on the infection of midgut cells has been observed when one or more of the components of the virion are absent. These components are called *Per Os* Infectivity proteins (PIFs) [39]. For these proteins, helper action must be in *cis*. The helper protein needs to be in the same particle [40].

A detailed analysis of the blocking point for CpGV-M infection of the CpRR1 colony of resistant insects has been published [10]. These authors demonstrated that the mechanism involved in resistance was not linked to midgut peritrophic membrane degradation. Moreover, in resistant CpRR1 insects challenged with the recombinant virus bacCpGVhsp-eGFP, based on CpGV-M, these authors detected a few cells in which expression of a marker carried by the virus was ongoing (see Figure 4d in Reference [10]), but generalized infection never occurred. Consequently, a model of generalized host cell/virus incompatibility has been proposed [10]. The results of our study reveal that the helper action of CpGV-R5 occurs when mixing OBs that had been produced independently in different hosts. This suggests the presence of a *trans*-acting effect that acts both at the level of the midgut and other larval tissues in resistant insects.

A major difference between multicapsid nucleopolyhedroviruses (MNPVs) and GVs should be considered. In MNPVs, OBs contain Multiple Occlusion Derived Virions (ODV), and each ODV contain a variable number of genomes. Defective virus genomes can be cooccluded in the same OB and probably co-enveloped in the same virion with replication-autonomous genomes, and can thus be transmitted together to a new host, the complete genome acting as a helper [41]. In GVs, OBs carry a single virion containing a single genome. The presence of both CpGV-M and CpGV-R5 genomes in a given larva relies on the ingestion of independent OBs.

The LD_50_ for CpGV-M in susceptible insects has been estimated at 1.2–5.0 OBs/larva [4]. In our tests, 50% mortality for CpGV-M in such susceptible insects is obtained with a concentration of 13.10 OB/µL. Consequently, at such a concentration, a larva would have eaten between 1.2 to 5 OBs. The lethal concentration observed for CpGV-R5 OBs in resistant insects is in the same range.

Accordingly, at low doses, most larvae that died from granulosis disease would have consumed only one or a few OBs. The highest probability for obtaining a mixed infection is when both viruses are present at the same frequency. For a larva eating 5 OBs (the upper estimation for LD_50_ on susceptible insects) randomly selected from a mixed population containing 50% CpGV-R5 and 50% CpGV-M; the probability of “had eaten both CpGV-M and CpGV-R5” is 0.84. In a population containing 90% CpGV-M and 10% CpGV-R5, for the same doses, only 38% of the insects would have eaten both virus genotypes. This probability increases when the number of OBs ingested increases. In our conditions, only when challenging hosts with a virus population containing 50% CpGV-R5 and 50% CpGV-M, almost all larvae will have consumed both virus genotypes (apart from insects treated with the lowest virus concentrations).

In addition, as in GVs, occlusion derived virions carry a single genome, and this helper effect for replication cannot occur unless a particular host cell is infected by at least two virions, one of which is a helper-genotype. Given these conditions, multiple infection of a midgut cell is probably a very rare event. Moreover, each infecting virus particle must be able to accomplish the whole cycle of infection, replication and cell-to-cell transmission within the infected insect. Accordingly, as the presence of CpGV-R5 in the host helps CpGV-M virions to infect, a possible explanation would involve a diffusible factor secreted by CpGV-R5-infected cells that renders non-infected cells permissive to CpGV-M infection. Alternatively, some cells could host a latent blocked infection of CpGV-M that becomes released if a CpGV-R5 superinfects.

Surprisingly, both virus genotypes were detected after replication of all experimental virus populations on RGV larvae, raising questions concerning the possible interactions between the viruses when infecting the larvae and the individual cells that should be explored further.

## 5. Conclusions

The use of mixed genotype virus isolates as the basis for biological insecticides allows efficient pest control while preserving virus diversity and probably reducing the impact of selective pressures on the pest population that favors resistance development. This would likely reduce the speed or the probability of generation of insect populations showing resistance to these virus isolates. Combining this approach with co-evolution of virus populations with the natural pest populations they are targeting will likely prevent development of new cases of resistance, making the biological control of this insect sustainable.

## Figures and Tables

**Table 1:** Pathogenicities, measured by Lethal Concentration (LC_50_ and LC_90_) of two viral isolates of Cydia pomonella granulovirus, CpGV-M and CpGV-R5 and mixtures of these viruses in different proportions in *C. pomonella* laboratory colonies that were susceptible (CpNPP) and resistant (R_GV_) to CpGV-M. Lines 1, 4, 6 and 8, are reference assays that have been published previously [33,34]. Bioassays were performed by diet surface contamination with neonate larvae. Mortality was scored at 7 days post-infection.

	Insect Strain	Composition of Virus Mixtures (%)		No. Controls	No. Insects Tested	Lethal Concentrations in OBs/µL (95% CI)		Slope ± SE	χ^2^
		CpGV-M	CpGV-R5			LC_50_	LC_90_		
1	CpNPP	100	0	257	529	13.10 (6.55–23.20)	223.10 (110.70–654.18)	1.04 ± 0.09	5.99
2		90	10	256	541	12.40 (4.92–25.42)	119.03 (52.17–616.73)	1.31 ± 0.12	11.95
3		50	50	257	518	10.69 (6.67–16.11)	94.12 (56.48–197.99)	1.36 ± 0.12	4.26
4		0	100	257	533	6.76 (2.59–13.37)	59.63 (27.54−278.55)	1.36 ± 0.13	11.42
5	R_GV_	100	0	476	1143	2.22 × 10^6^ (1.19 × 10^6^–5.67 × 10^6^)	-	0.50 ± 0.07	10.6
6		90	10	215	817	201.78 (139.95–280.83)	2.35 × 10^3^ (1.51 × 10^3^–4.23 × 10^3^)	1.20 ± 0.09	5.46
7		50	50	354	989	16.45 (8.41–29.07)	311.55 (151.53–938.21)	1.00 ± 0.06	16.35
8		0	100	176	369	22.43 (13.73–34.36)	410.67 (240.16–846.43)	1.02 ± 0.11	3.60

**Table 2:** Results of the Independent Joint Action test for mixed virus populations M50-R50 and M90-R10 compared to genotypically homogeneous populations of Cydia pomonella granulovirus CpGV-M and CpGV-R5.

Insect Strain	Composition of Virus Mixtures (%)		Chi-Square	Degrees of Freedom	P
	CpGV-M	CpGV-R5			
CpNPP	50	50	91.002	6	<0.01
	90	10	8.98	6	>0.05
R_GV_	50	50	40.276	6	<0.01
	90	10	4.581	6	>0.05
